# Kinesiophobia in a Patient With Postoperative Midshaft Fracture: A Case Report of Its Impact on Rehabilitation in a 16-Year-Old Girl

**DOI:** 10.7759/cureus.11333

**Published:** 2020-11-05

**Authors:** Madhuri Wane, Waqar M Naqvi, Laukik Vaidya, Kiran Kumar

**Affiliations:** 1 Occupational Therapy, Datta Meghe Institute of Medical Sciences, Wardha, IND; 2 Community Physiotherapy, Datta Meghe Institute of Medical Sciences, Wardha, IND; 3 Physiotherapy, Datta Meghe Institute of Medical Sciences, Wardha, IND

**Keywords:** kinesiophobia, rehabilitation, midshaft femur fracture and open reduction internal fixation

## Abstract

Kinesiophobia is an irrational and debilitating fear of physical movement and activity resulting from a feeling of vulnerability to painful injury or re-injury. According to the concept of avoidance of fear, pain is interpreted as threatening, which can trigger pain-related fears and anxiety leading to avoidance behavior. Avoidance action involves a process/period characterized by a person stepping back from undertaking daily tasks like exercise, socializing, and work, which increases the intensity of the painful experience. In hospital settings, kinesiophobia needs to be resolved to ensure a positive result in rehabilitation interventions. The femur is the lower extremity's primary weight-bearing bone. Early fracture fixation in the shaft of the femur allows for early mobilization, thereby reducing the risk of hip and knee stiffness as well as quadriceps and hamstring wasting.

In this report, we present the case of a 16-year-old girl with an alleged history of fall who was admitted to Acharya Vinobha Bhave Rural Hospital (AVBRH), Datta Meghe Institute of Medical Sciences (DMIMS) Deemed To Be University (DU), Wardha, India, with primary complaints of pain and swelling over the left thigh. She was diagnosed with a left midshaft femur fracture. An open reduction internal fixation (ORIF) femur interlock nailing was performed to stabilize the fracture, and she was referred to physiotherapy after surgery for further management. The comprehensive rehabilitation program was helpful in alleviating the severe kinesiophobia in the patient, and she was able to resume her activities of daily living (ADLs) independently.

## Introduction

Kinesiophobia was defined by the people who coined the term as “an irrational, and debilitating fear of physical movement and activity resulting from a feeling of vulnerability to painful injury or re-injury" [1]. According to the concept of avoidance of fear, pain is perceived as threatening, which can trigger pain-related fears and anxiety leading to avoidance behavior. Avoidance action constitutes a behavior pattern where a person stays away from daily tasks like exercise, socializing, and work, which in turn aggravates the experience of pain [2]. Kinesiophobia causes a vicious cycle of pain-avoidance and fear in people who experience it, which increases their disability perception and contributes to disuse syndrome among them. Movement anxiety and other psychological and emotional aspects are strongly connected to pain, and modulating them plays a critical role in the care process [3].

Skeletal injury in blunt trauma remains one of the primary causes of long-term impairment among youth [4]. Falls and road traffic accidents are the major causes of femoral shaft fractures [5]. The femur is the lower extremity's primary weight-bearing bone. Fracture of the femoral shaft generally occurs with high-energy injuries, which can be linked to multisystem. Early fracture fixation in the shaft of the femur allows for early mobilization, thereby reducing the risk of hip and knee stiffness as well as quadriceps and hamstring wasting. Intramedullary nail restores the shaft length and enables early load-bearing [6].

## Case presentation

We present the case of a 16-year-old girl with an alleged history of fall, who was admitted to Acharya Vinobha Bhave Rural Hospital (AVBRH), Datta Meghe Institute of Medical Sciences (DMIMS) Deemed To Be University (DU), Wardha, Maharashtra, India, with the complaints of pain and swelling over the left thigh. She had no history of loss of consciousness, vomiting, or seizures, and had a Glasgow Coma Scale of 15/15 following the event; necessary investigations confirmed that she had suffered a left midshaft femur fracture. An open reduction internal fixation (ORIF) femur interlock nailing was done. She was then referred to physiotherapy for further management (Table 1).

**Table 1:** Timeline of events

A timeline of events related to the patient's fall and treatment	
Events	Date
Fall from the second floor of her house	March 11, 2020
Diagnosed with midshaft femur fracture on the left side	March 12, 2020
Open reduction internal fixation done for midshaft femur fracture on the left side	March 12, 2020
Referred to physiotherapy for further management	March 14, 2020

Clinical findings

She was examined in a supine position with both shoulders at the same level. Following this, the left leg was abducted and slightly externally rotated, and knee extended with pillow support below knee and leg and ankle where ankle was slightly plantar flexed. The swelling was found to be present on the left mid-thigh and operative site (Tables 2-3). On palpation, there was a rise in local temperature with non-pitting edema. She also had extreme kinesiophobia with a Tampa Scale score of 67 and a visual analog scale (VAS) score of 10/10 [7].

**Table 2:** Isometric strength indicators

Indicators of isometric strength		
Muscles	Right	Left
Hip		
Flexors	Good	Poor
Extensors	Good	Poor
Abductors	Good	Poor
Adductors	Good	Poor
Knee		
Flexors	Good	Poor
Extensors	Good	Poor
Ankle		
Plantar flexors	Good	Poor
Doris flexors	Good	Poor
Invertors	Good	Poor
Evertors	Good	Poor

**Table 3:** Range of motion

Joint	Right active	Right passive	Left active	Left Passive	Limitations
Hip					
Flexion	0-115^0^	0-125^0^	NA	NA	Unable to perform due to pain
Extension	0-110^0^	0-115^0^	NA	NA	Unable to perform due to pain
Abduction	0-40^0^	0-45^0^	NA	NA	Unable to perform due to pain
Adduction	40-0^0^	45-0^0^	NA	NA	Unable to perform due to pain
Medial rotation	0-45^0^	0-45^0^	NA	NA	Unable to perform due to pain
Lateral rotation	0-45^0^	0-45^0^	NA	NA	Unable to perform due to pain
Knee					
Flexion	0-125^0^	0-130^0^	NA	NA	Unable to perform due to pain
Extension	125-0^0^	135-0^0^	NA	NA	Unable to perform due to pain
Ankle					
Plantar flexion	0-45^0^	0-50^0^	NA	NA	Unable to perform due to pain
Doris flexion	0-15^0^	0-20^0^	NA	NA	Unable to perform due to pain
Inversion	0-30^0^	0-35^0^	NA	NA	Unable to perform due to pain
Eversion	0-20^0^	0-25^0^	NA	NA	Unable to perform due to pain

Medical management

**Figure 1:**
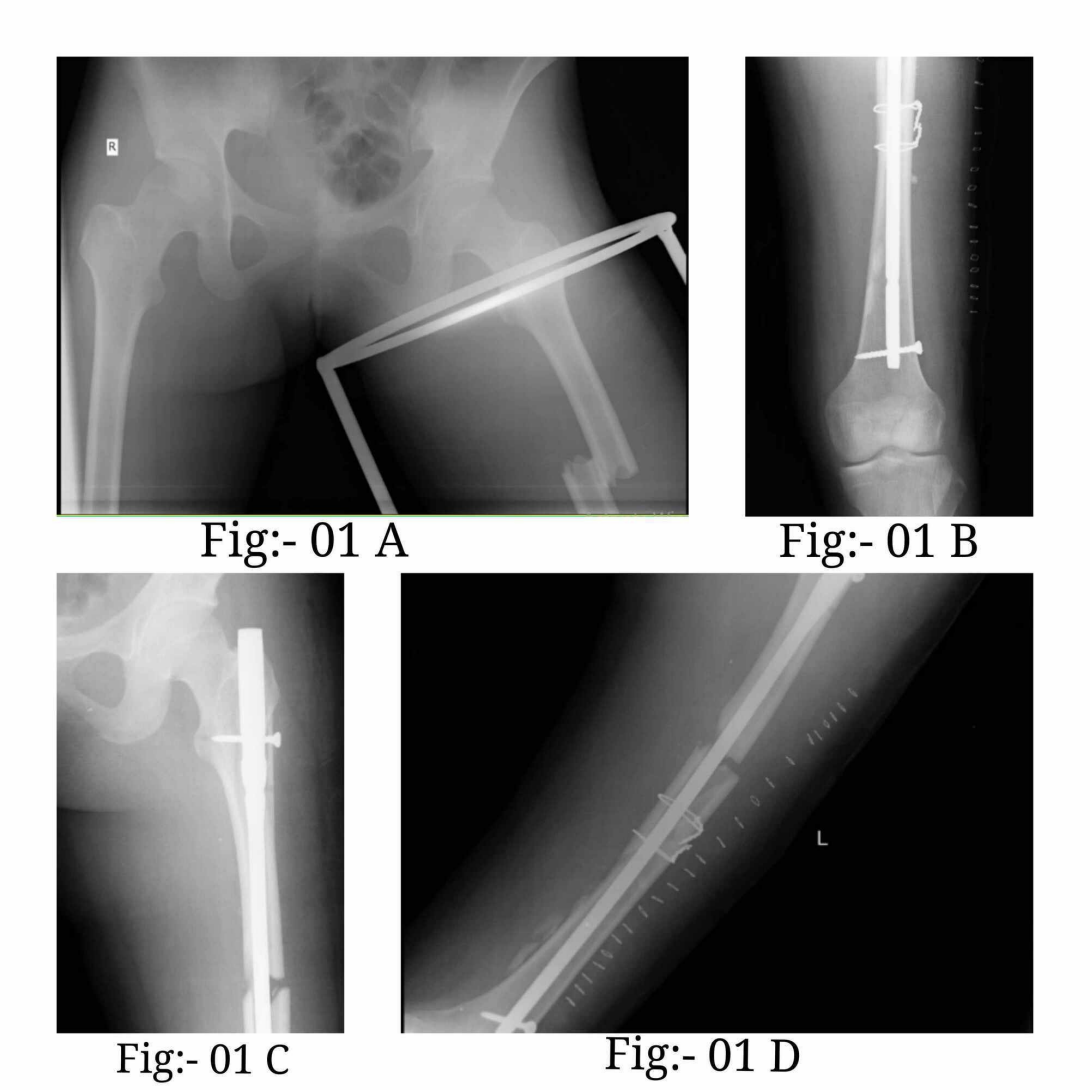
X-ray of the patient A-D: an ORIF femur interlock nailing with one proximal screw and one distal screw locking was done. Stainless steel wire was also used in the reduction for the patient ORIF: open reduction internal fixation

**Figure 2:**
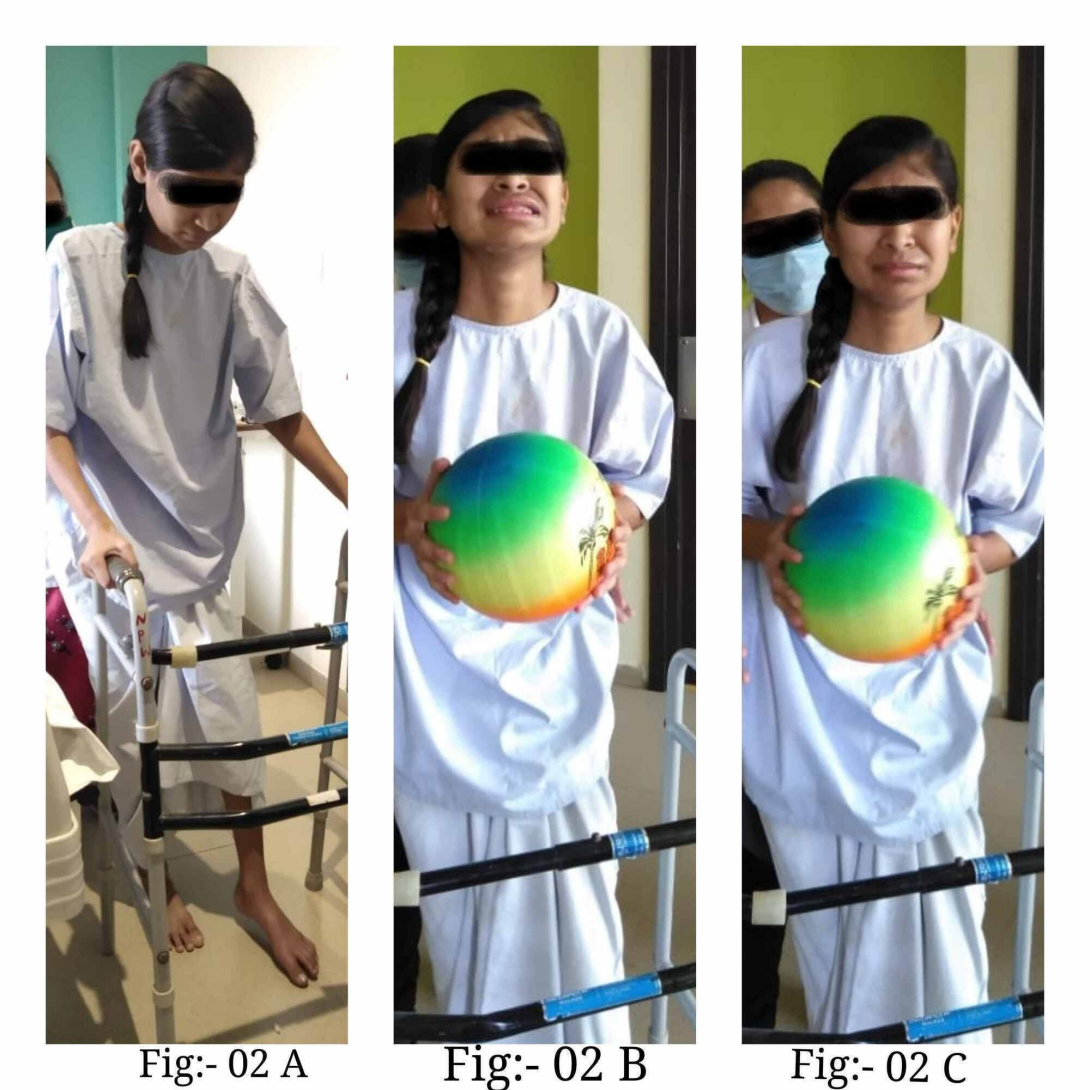
Postoperative rehabilitation A-C: the postoperative rehabilitation procedure was spread over four phases, which involved exercises with a walker and weight-bearing activities apart from counseling and other tasks

An ORIF femur interlock nailing with one proximal screw and one distal screw locking was done. Stainless steel wire was also used in the reduction procedure (Figure 1A-1D). Figure 2A-2C presents images that depict the patient's postoperative rehabilitation process.

Goals

Postoperative

The short-term goals were to prevent respiratory complications, reduce pain and edema, maintain and increase joint range of motion and strength, promote early mobility, avoid pressure sores, encourage walking (non-weight bearing), and enable the patient to carry out ADLs independently [8-10].

**Figure 3:**
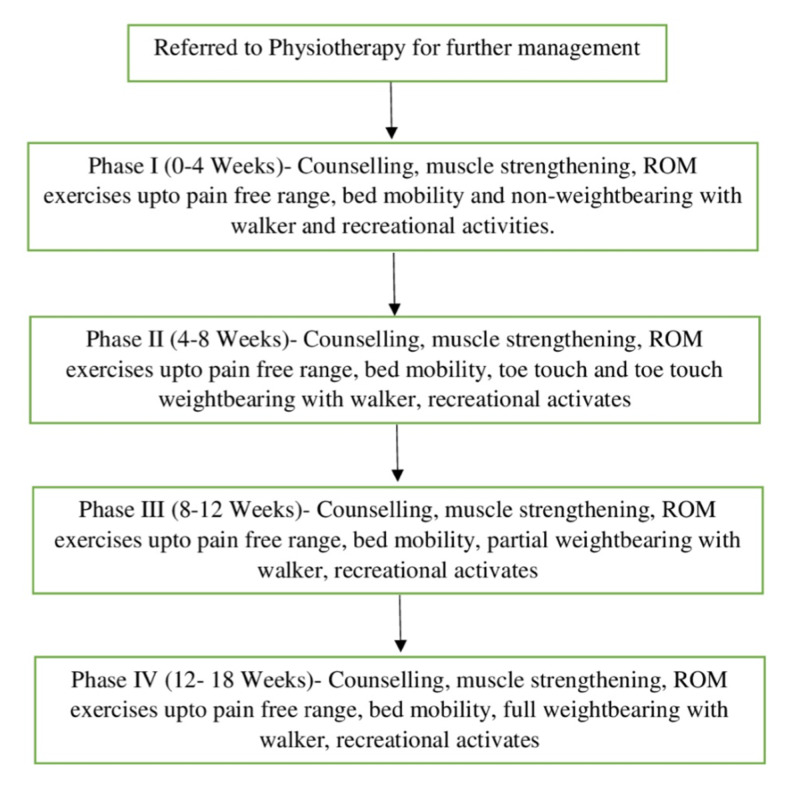
Timeline of therapeutic management ROM: range of motion

The long-term goals were to promote independent walking with and without the walker (partial/full weight-bearing), static and dynamic balance, to promote indoor and outdoor mobility, independent ADLs, and ergonomics (Figure 3).

Therapeutic management

Phase I (Zero to Two Weeks)

Phase I exercises revolved around counseling, knee and hip strengthening, non-weight bearing, and mobility of joints. Active range of motion, knee flexion exercises, and lower limb isometrics were initiated immediately after the surgery. The management of effusion and edema was carried out by frequent cryotherapy and limb elevation. Active control of the musculature of the knee extensor and the hip abductor were concentrated in immediate strengthening. Open chain exercises such as simple hip abduction, extension, and flexion exercises were done for strengthening a non-weight-bearing position. Throughout these exercises, the emphasis was placed on sustaining a powerful contraction of quadriceps with the leg in a completely extended posture. The knee extension was performed actively in a sitting position, without weight (open chain). It would also aid to avoid an independent quadriceps operation. This continued with non-weight-bearing with a walker.

Phase II (Two to Six Weeks)

In phase II, many aspects of the phase I regimen were continued as needed. Weight-bearing with a walker proceeded further to encourage toe contact (10% weight). Phase II interventions involved the advancement of phase I exercises to improve weight-bearing tasks, some gait retraining, recreational activities, and strength-conditioning initiation.

Strengthening advanced toward further weight-bearing activities until the patient began to display the ability to bear weight. More non-weight-bearing activities like knee extension with resistance bands ranging from 900 of flexion to 300 of flexion were started. Hip-strengthening progressed further by the introduction of exercises like abduction and flexion of the hip in a standing position. Training activities in phase I was repeated with increased resistance and more frequency.

Phase III (6-12 Weeks)

In order to improve mobility, balance, proprioception, and agility training, the focus of the exercises was on increasing the power of the lower limb by partial weight-bearing activities during phase III.

Power progression, gait normalization, and an eventual shift to performing desired activities were concentrated on. The increase in the intensity of the exercise implemented by enhanced resistance with progressive resisted exercises (PRESs) focused on strengthening the patient's condition. Furthermore, the advancement of weight-bearing to a level of 50% with or without an assistive tool, and strengthening exercises like step-ups on a single leg, were also initiated. Emphasis was continued to be placed on impairments that caused functional limitations, in particular the extension of knee and abduction of hip activities. The static balance development was done by ensuring that stable platform activities were advanced to make stable and unstable surfaces more dynamic, and training was enhanced by stressing the need to practice a more normal gait pattern. Along with backward-walking, gait-training exercises progressed to side-stepping activities with necessary help.

Phase IV (12-18 Weeks)

Throughout phase IV, in addition to improving mobility, coordination, fitness conditioning, and proprioception, the focus was also on exercises to improve full-weight strength bearing functions in the lower limb.

Strength improvement, gait normalization, and an eventual shift to desired activities were continued to be pursued in this stage. In terms of reinforcement, improvement in the intensity of the initiated exercises by enhanced resisted PRESs was also implemented. Similarly, for weight improvement of up to 100% with or without the walker, individuals usually pursue tasks like step-up, half lunges, and mini squat. For more closed-chain exercises like lateral walking with resistance, the strengthening of hip abduction was also advanced. Functional restricting impairments, targeted specifically by knee extension and hip abduction practices, were also an area of focus. Balancing and proprioception training progressed as single-leg tasks when full weight-bearing was carried out with or without the aids. Exercises with a solid platform with the transition of static balance to more active single-leg exercises on stable and precarious surfaces along with rehabilitation were implemented with an emphasis on practicing a more normal gait pattern.

Limitations

Kinesiophobia leads to a vicious cycle of pain-avoidance and fear, which severely affects the ability of the patient to perform any movement and activities that involve the operated limb; hence, the initial range of motion exercises was difficult.

## Discussion

We discussed the case of a young girl who suffered an alleged fall and subsequent midshaft femur for which she was operated on with ORIF to stabilize the fracture. Rehabilitation objectives were formulated with regards to her kinesiophobia (Tampa Scale Score of 34), beginning from mild exercises to strengthening and total weight-bearing walker ambulation. All the exercises were performed three times a day and consisted of 10 sets each; ice packs were placed around the patient's leg to restrict movements that would cause pain during the counseling session.

Since she had a fall, the patient developed a fear that any movement of her body would injure her further, and this was driven by an unreasonable belief in her susceptibility to injury. In patients with kinesiophobia, a sense of fear is generated regarding excessive movement because of the belief that they are vulnerable to injury, which leads to declining physical activity and musculoskeletal disorders correlated with pain.

Pain in such patients is related to kinesiophobia. Physical activity is essential for the health of the bone and the body in general, and patients should be educated and informed about osteoporosis and the importance of the physical activity to overcome the condition, as there is a moderate yet significant association of kinesiophobia with mobility and balance. The pain perspective activates kinesiophobia, which eventually contributes to behavior avoidance, which in turn can influence functional performance as well as lower limb function. To avoid this behavior, we concentrated on active movements, but weight-bearing was delayed due to the presence of this condition in the patient.

The underlying health aspects following injuries as well as the rehabilitation protocol must be considered [8]. Patients with serious soft tissue injuries, including open fractures, crush injuries, or even extreme contusions of the thigh, may raise a medical problem that would require modifications to the rehabilitation process. Flexibility with all these circumstances would be essential; however, a goal-based advancement of the therapy process should be included with every attempt, particularly in ensuring that progression to weight-bearing activity should be made as soon as possible since it really pushes the recovery progress. The goals were modified to fit the requirement by our team with regards to the injury and the associated fear in our patient.

The emphasis should be on immediate weight-bearing and progress in the gait-training; the extent of muscular weakness in quadriceps femoris is a common cause for concern in femoral fracture, with and without the influence of surgery [9]; the identified deficiency in strength may also be due to the muscle damage suffered due to injury, and it has been shown that quadriceps femoris muscle weakness is related to the evaluated displacement of the fracture. With our patient, we emphasized on quadriceps while early weight-bearing was delayed due to kinesiophobia.

## Conclusions

In this case study, we discussed a 16-year-old female with post-surgical midshaft femur fracture who had severe kinesiophobia, which impacted her rehabilitation. A rehabilitation plan consisting of goal-oriented occupational therapy, physiotherapy, and psychological counseling reduced the pain related to kinesiophobia in our patient and improved her functional independence. With the aid of constant therapy, she eventually resumed her ADLs independently.
